# Supplementary material for: Fast site-to-site electron transfer of high-entropy alloy nanocatalyst driving redox electrocatalysis
Source: Nat Commun. 2020 Oct 28;11:5437. doi: 10.1038/s41467-020-19277-9 (PMC7595151; doi:10.1038/s41467-020-19277-9)
Supplement: Supplementary file 1 — Supporting Information [file 41467_2020_19277_MOESM1_ESM.pdf]

Supporting Information for

Fast Site-to-site Electron Transfer of High-entropy Alloy Nanocatalyst Driving Redox Electrocatalysis

Hongdong Li[1], Yi Han[1], Huan Zhao[1], Wenjing Qi[4], Dan Zhang[1,2], Yaodong Yu[1], Wenwen Cai[1], Shaoxiang Li[2], Jianping Lai[1,], Bolong Huang[3,*] and Lei Wang[1,2,*]*

[1]Key Laboratory of Eco-chemical Engineering, Key Laboratory of Optic-electric Sensing and Analytical Chemistry of Life Science, Taishan Scholar Advantage and Characteristic Discipline Team of Eco Chemical Process and Technology, College of Chemistry and Molecular Engineering, Qingdao University of Science and Technology, Qingdao 266042, P. R. China

E-mail: inorchemwl@126.com; jplai@qust.edu.cn

[2]Shandong Engineering Research Center for Marine Environment Corrosion and Safety Protection, College of Environment and Safety Engineering, Qingdao University of Science and Technology, Qingdao 266042, P. R. China

[3]Department of Applied Biology and Chemical Technology, The Hong Kong Polytechnic University, Hung Hom, Kowloon, Hong Kong SAR, China

E-mail: bhuang@polyu.edu.hk

[4]College of Chemistry, Chongqing Normal University, Chongqing 401331, P. R. China

Figures

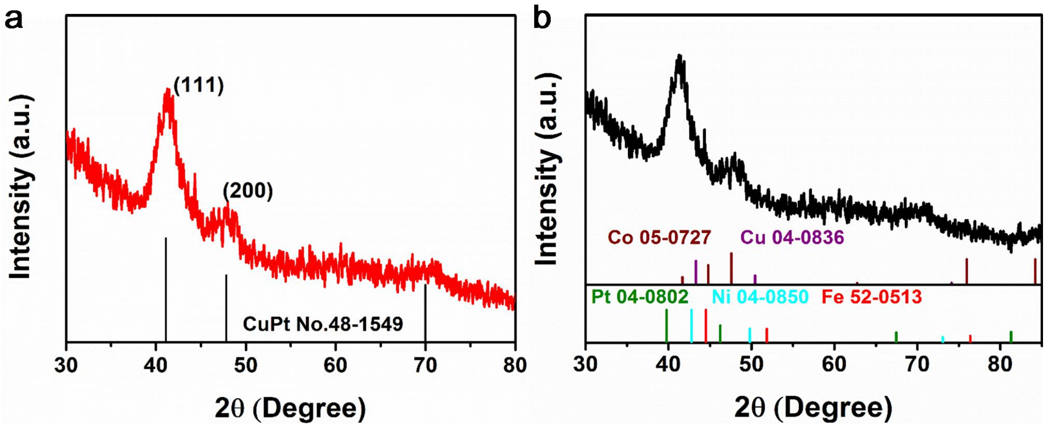

Supplementary Figure 1. XRD patterns of $Pt_{18}Ni_{26}Fe_{15}Co_{14}Cu_{27}$ nanoparticles.

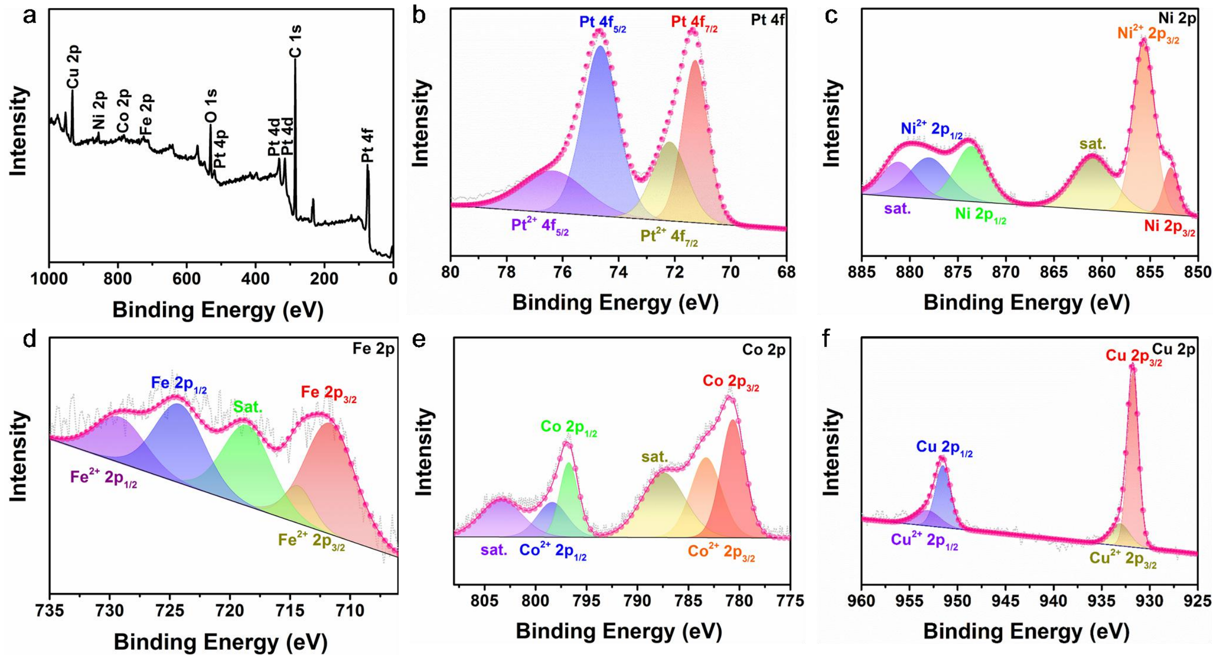

Supplementary Figure 2. XPS compositional analysis of $Pt_{18}Ni_{26}Fe_{15}Co_{14}Cu_{27}$ nanoparticles. (a) XPS survey spectrum. (b) Pt 4f XPS spectrum. (c) Ni 2p XPS spectrum. (d) Fe 2p XPS spectrum. (e) Co 2p XPS spectrum. (f) Cu 2p XPS spectrum.

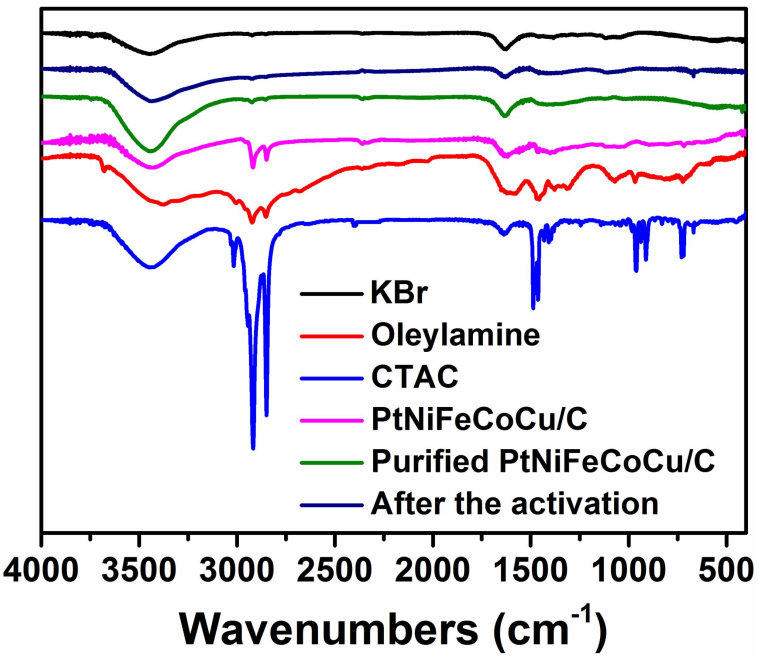

Supplementary Figure 3. FTIR spectra of the pure KBr, pure oleylamine, pure CTAC, PtNiFeCoCu/C, the purified PtNiFeCoCu/C and after the activation before the electrocatalysis tests.

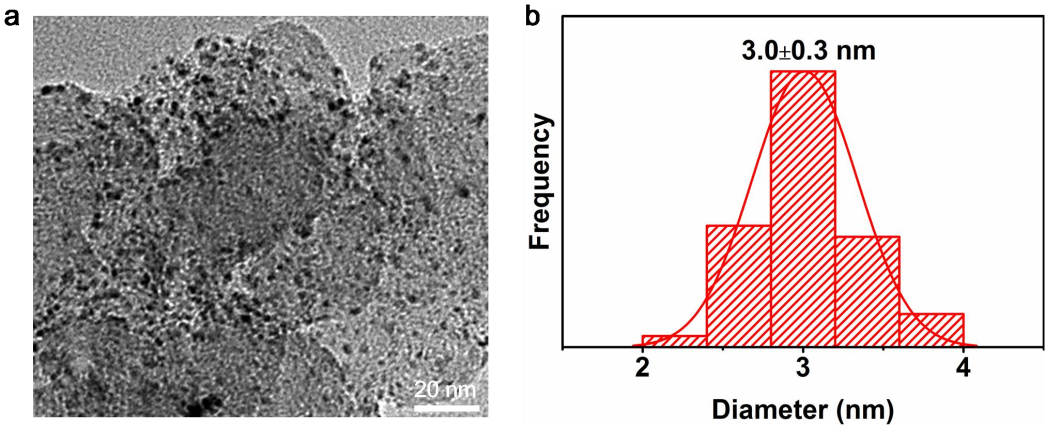

Supplementary Figure 4. TEM image and size distribution of the Pt/C catalyst. (a) The TEM image of Pt/C catalyst. (b) The histogram of the diameter of Pt/C catalyst.

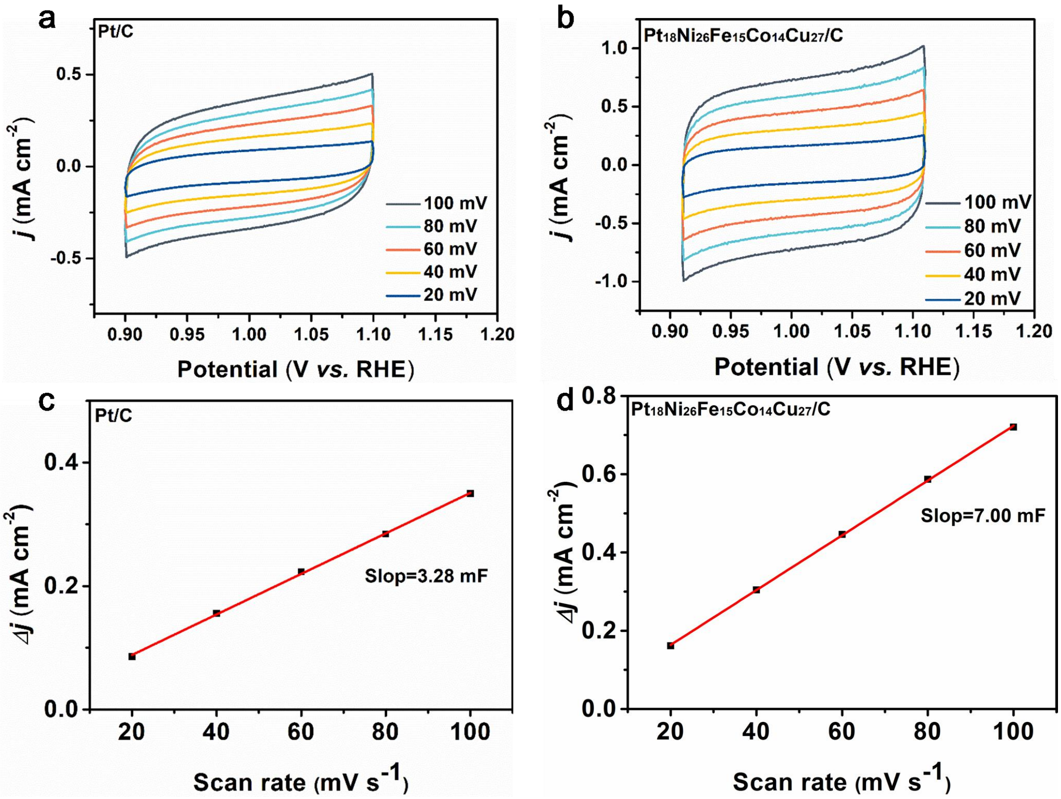

Supplementary Figure 5. Electrocatalytic property evaluation. CV curves of (a) Pt/C and (b) $Pt_{18}Ni_{26}Fe_{15}Co_{14}Cu_{27}/C$ in the double layer region at scan rates of 20, 40, 60, 80 and 100 mV s^{-1} in 1.0 M KOH. (c) Pt/C and (d) $Pt_{18}Ni_{26}Fe_{15}Co_{14}Cu_{27}/C$, current density as a function of scan rate derived from (a) and (b) at 1.0 V (*vs.* RHE), respectively.

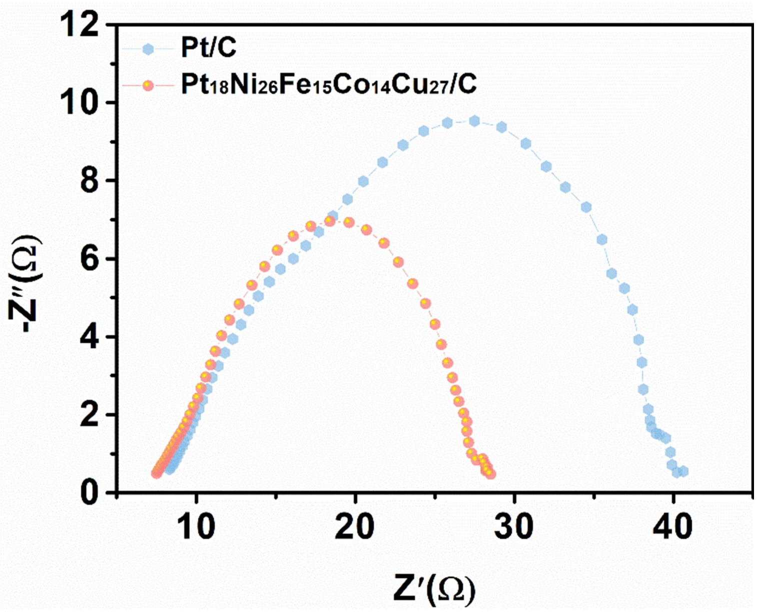

Supplementary Figure 6. The Nyquist plots of $Pt_{18}Ni_{26}Fe_{15}Co_{14}Cu_{27}$/C and Pt/C at -50 mV *vs.* RHE.

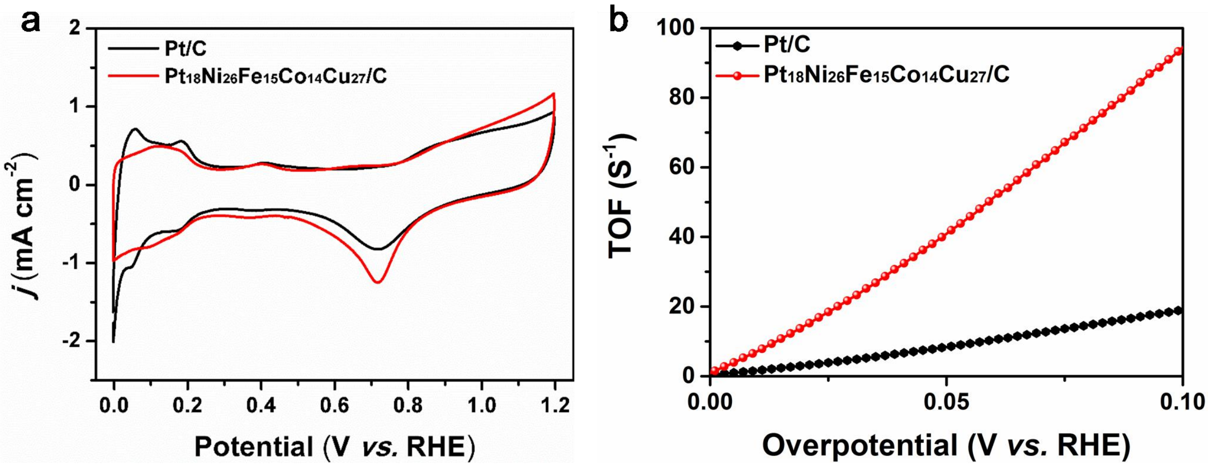

Supplementary Figure 7. Electrocatalytic property evaluation. (a) CV curves of $Pt_{18}Ni_{26}Fe_{15}Co_{14}Cu_{27}$/C and Pt/C catalysts in 0.5 M H_2SO_4. (b) The potential dependent TOF curves of $Pt_{18}Ni_{26}Fe_{15}Co_{14}Cu_{27}$/C and Pt/C.

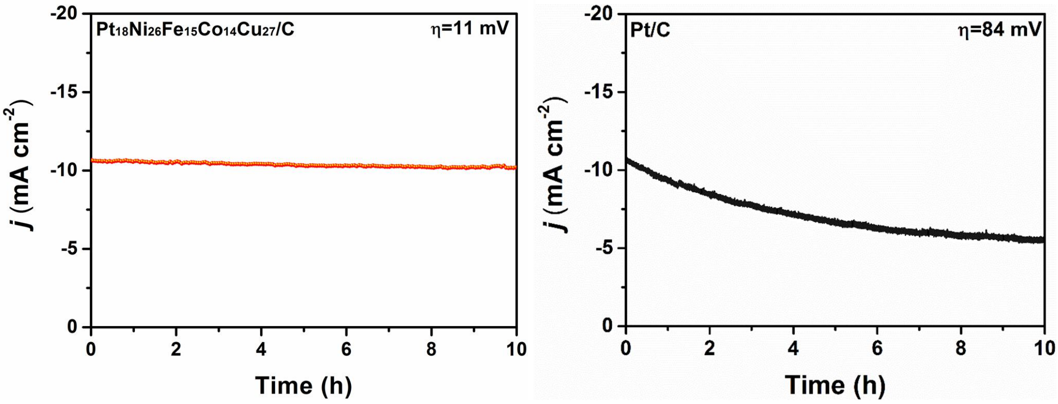

Supplementary Figure 8. Electrocatalytic durability tests. Chronoamperometric measurement curves of (a) $Pt_{18}Ni_{26}Fe_{15}Co_{14}Cu_{27}/C$ catalyst and (b) Pt/C catalyst for HER.

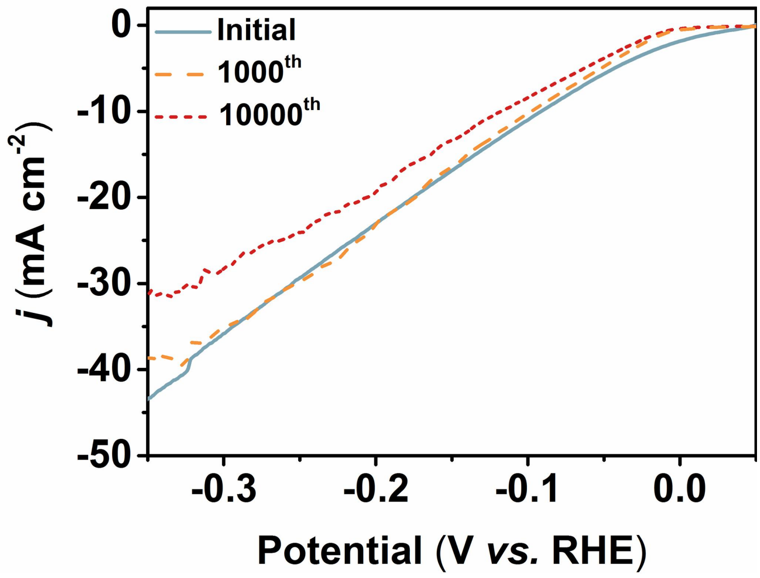

Supplementary Figure 9. HER polarization curve (geometrical area) for Pt/C with different CV cycles.

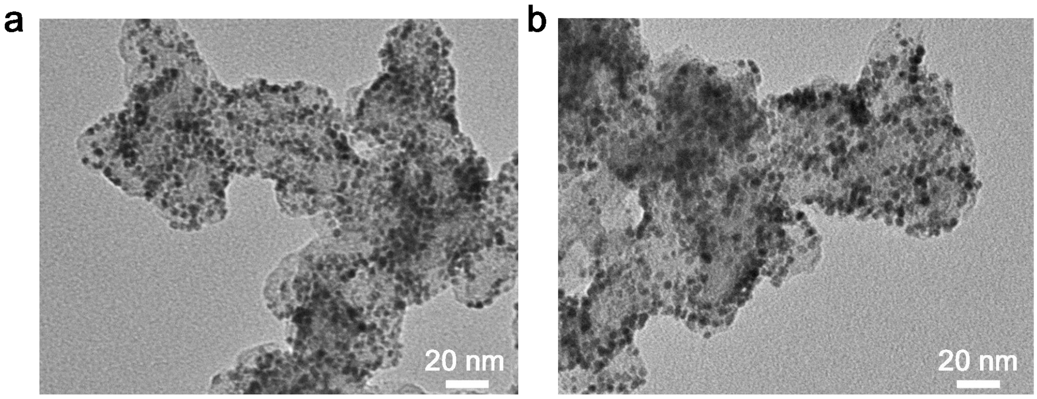

Supplementary Figure 10. TEM images of nanocatalysts before and after HER. The TEM images of $Pt_{18}Ni_{26}Fe_{15}Co_{14}Cu_{27}/C$ before (a) and after (b) the stability test for HER.

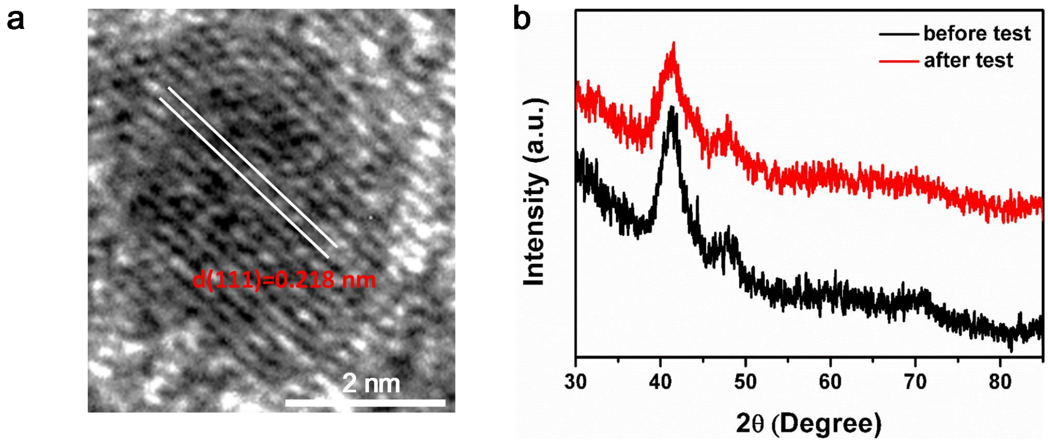

Supplementary Figure 11. Characterization of the $Pt_{18}Ni_{26}Fe_{15}Co_{14}Cu_{27}/C$ catalyst after stability test for HER. (a) HRTEM image of $Pt_{18}Ni_{26}Fe_{15}Co_{14}Cu_{27}/C$. (b) XRD patterns of $Pt_{18}Ni_{26}Fe_{15}Co_{14}Cu_{27}/C$.

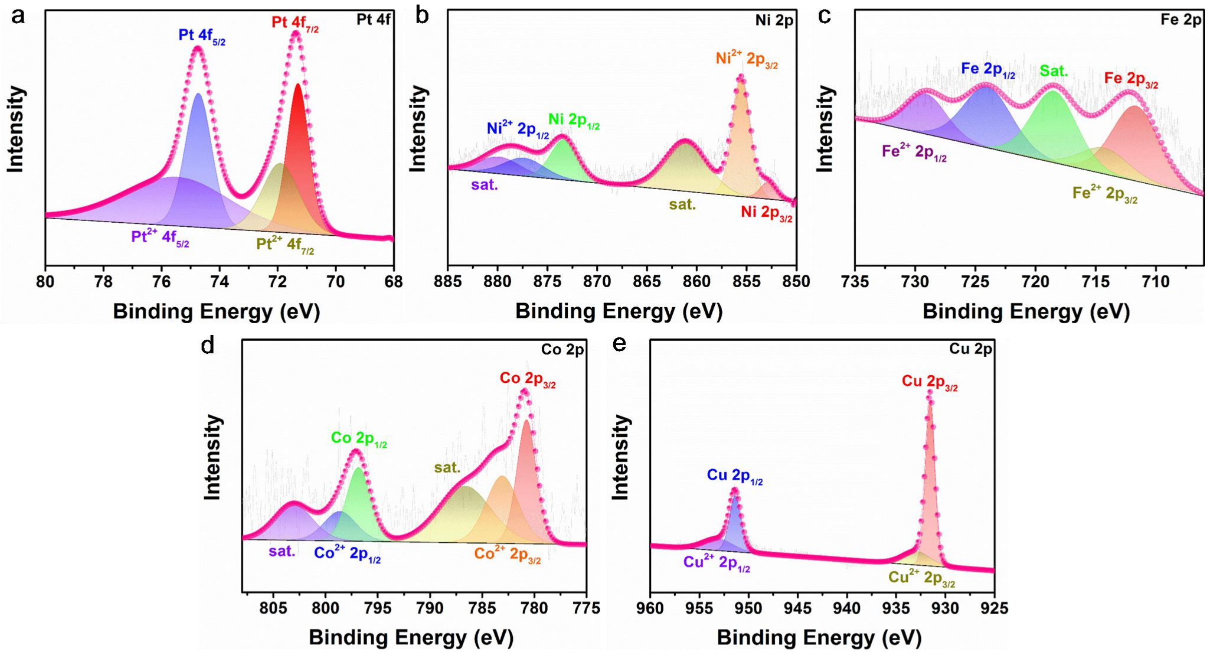

Supplementary Figure 12. XPS analysis of $Pt_{18}Ni_{26}Fe_{15}Co_{14}Cu_{27}/C$ catalyst after HER. (a) Pt 4f XPS spectrum. (b) Ni 2p XPS spectrum. (c) Fe 2p XPS spectrum. (d) Co 2p XPS spectrum. (e) Cu 2p XPS spectrum.

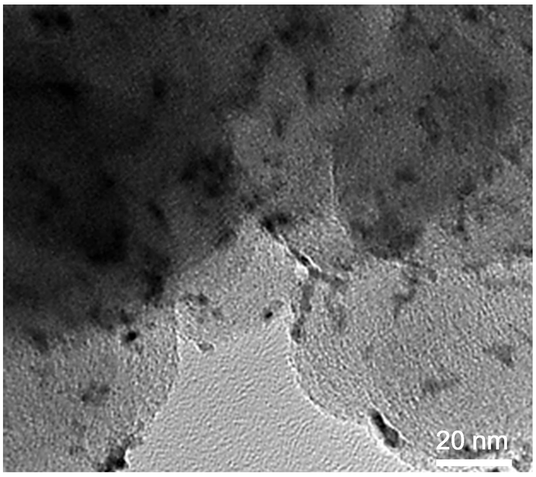

Supplementary Figure 13. TEM image. The TEM image of Pt/C after the stability test for HER.

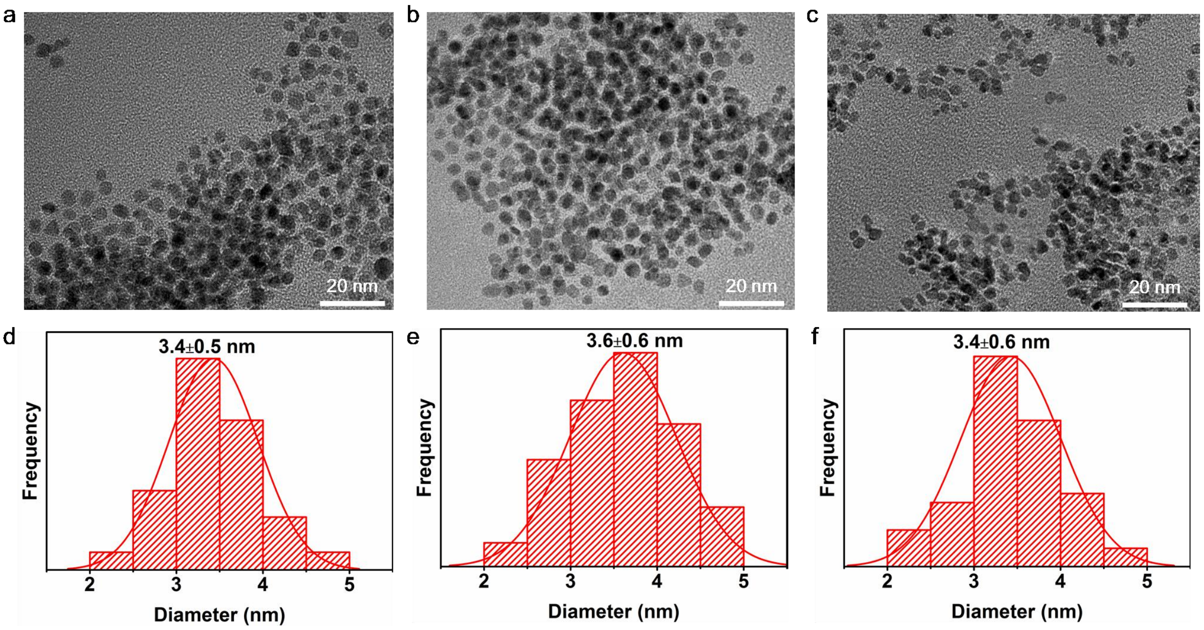

Supplementary Figure 14. The TEM images and size distribution of different PtNiFeCoCu nanoparticles. (a) The TEM image and (d) histogram of the diameter of $PtNiFeCoCu_{26}$ NPs. (b) The TEM image and (e) histogram of the diameter of $Pt_{21}Ni_{27}Fe_{19}Co_{17}Cu_{16}$ NPs. (c) The TEM image and (f) histogram of the diameter of $Pt_{15}Ni_{24}Fe_{13}Co_{13}Cu_{35}$ NPs.

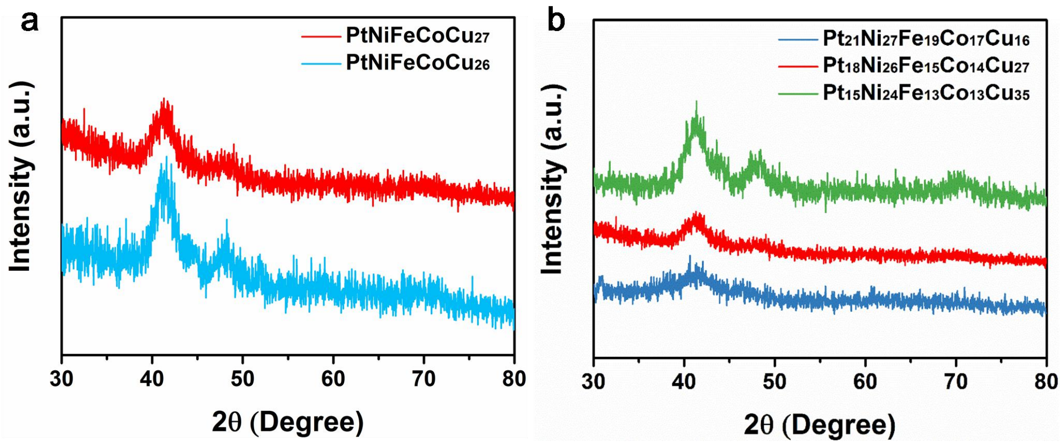

Supplementary Figure 15. XRD patterns. (a) XRD patterns of $PtNiFeCoCu_{27}$ NPs and $PtNiFeCoCu_{26}$ NPs. (b) XRD patterns of different PtNiFeCoCu NPs.

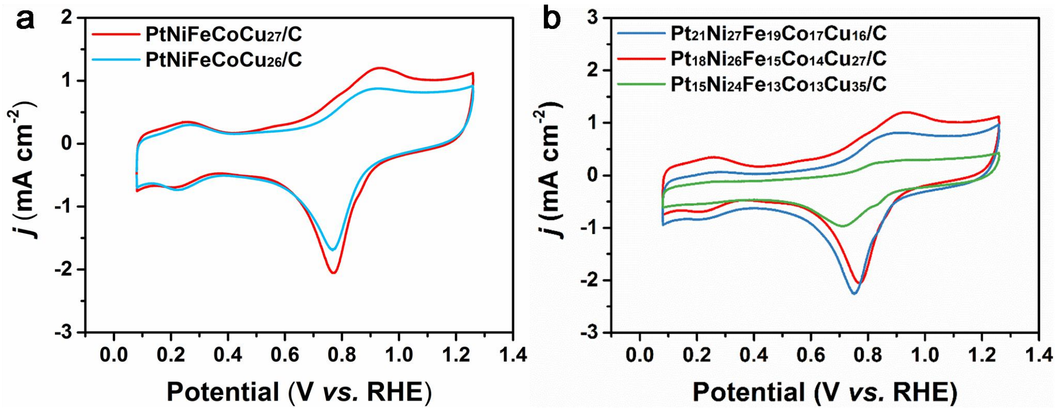

Supplementary Figure 16. Electrocatalytic property evaluation. (a) CV curves of the $PtNiFeCoCu_{27}/C$ and $PtNiFeCoCu_{26}/C$ and (b) CV curves of the $Pt_{21}Ni_{27}Fe_{19}Co_{17}Cu_{16}/C$, $Pt_{18}Ni_{26}Fe_{15}Co_{14}Cu_{27}/C$ and $Pt_{15}Ni_{24}Fe_{13}Co_{13}Cu_{35}/C$ in 1 M KOH electrolyte.

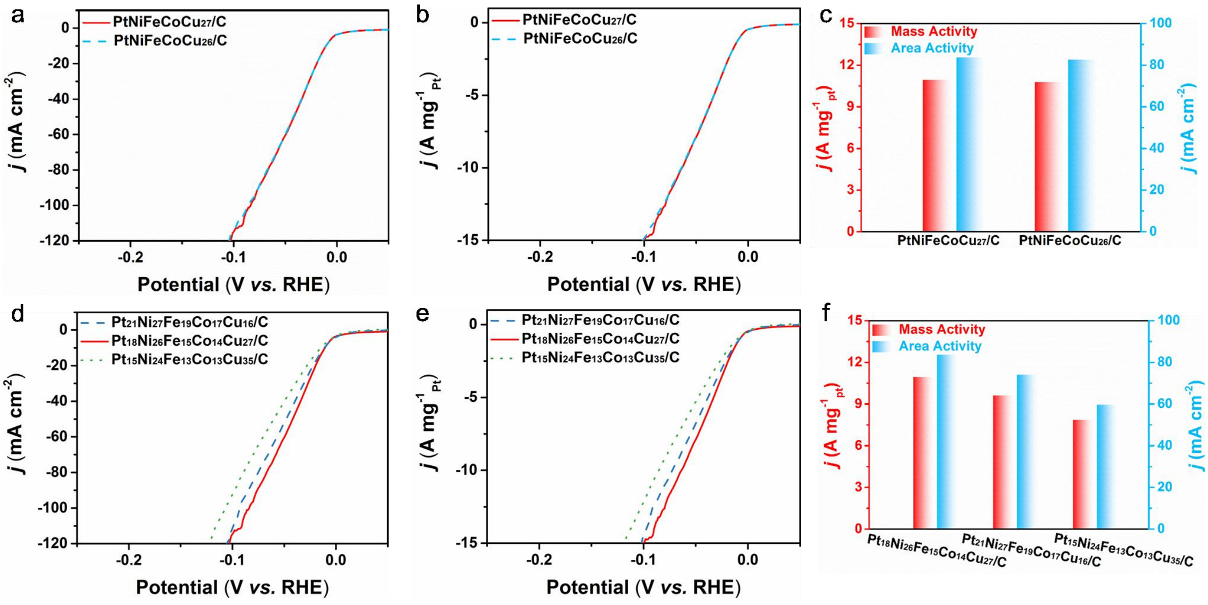

Supplementary Figure 17. Electrocatalytic performance of the $PtNiFeCoCu_{26}/C$, $Pt_{21}Ni_{27}Fe_{19}Co_{17}Cu_{16}/C$, $Pt_{18}Ni_{26}Fe_{15}Co_{14}Cu_{27}/C$ and $Pt_{15}Ni_{24}Fe_{13}Co_{13}Cu_{35}/C$ for HER in 1 M KOH electrolyte. (a) and (d) HER polarization curves (geometrical area). (b) and (e) Pt mass loading normalized (mass activity) LSV curves. (c) and (f) Comparison of area activity and mass activity values for HER at -70 mV *vs.* RHE.

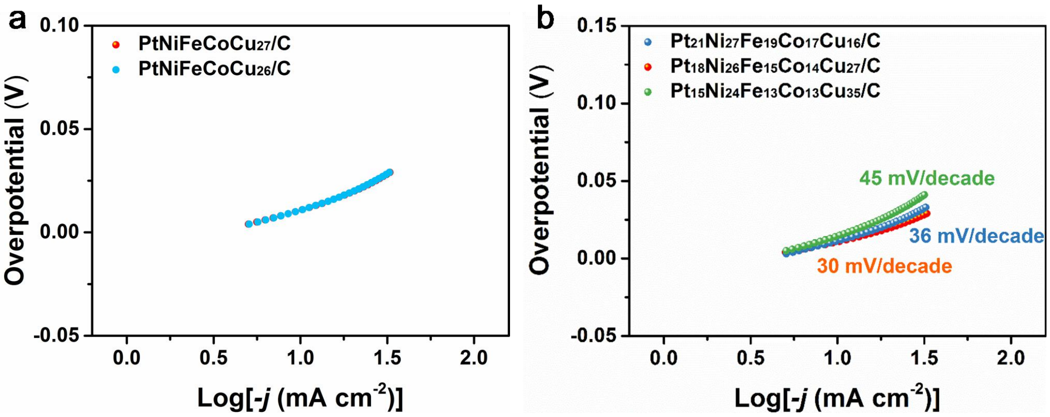

Supplementary Figure 18. Electrocatalytic property evaluation. (a) Tafel slopes of the PtNiFeCoCu$_{26}$/C and PtNiFeCoCu$_{27}$/C. (b) Tafel slopes of the Pt$_{21}$Ni$_{27}$Fe$_{19}$Co$_{17}$Cu$_{16}$/C, Pt$_{18}$Ni$_{26}$Fe$_{15}$Co$_{14}$Cu$_{27}$/C and Pt$_{15}$Ni$_{24}$Fe$_{13}$Co$_{13}$Cu$_{35}$/C.

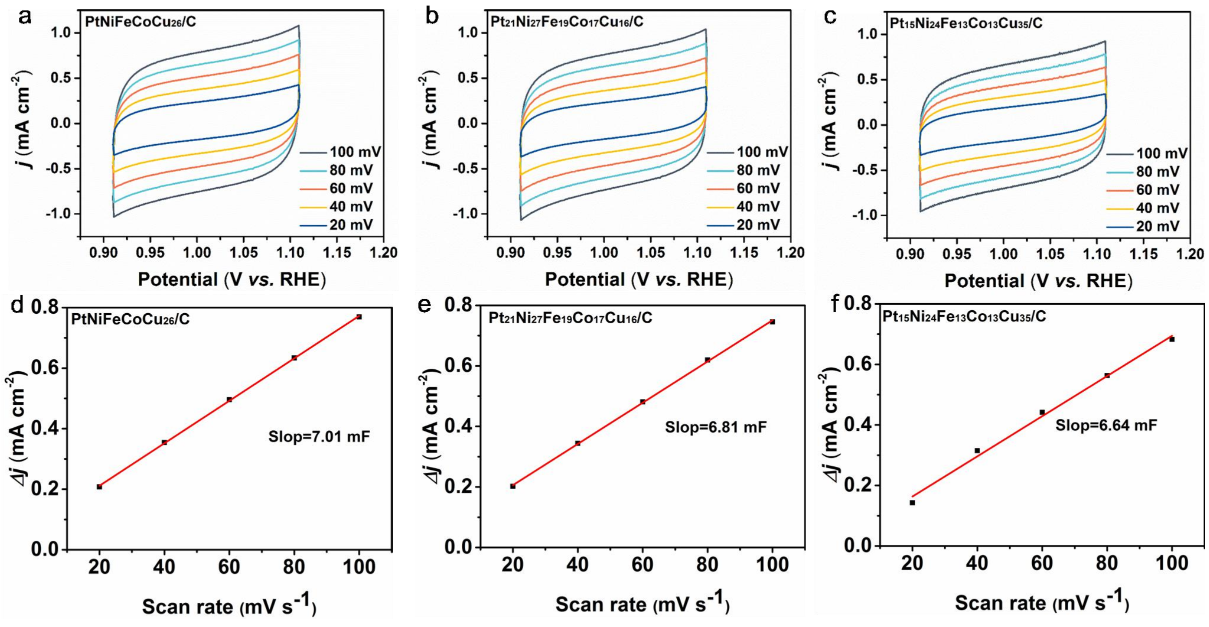

Supplementary Figure 19. Electrocatalytic property evaluation. (a)-(c) CV curves in the double layer region at scan rates of 20, 40, 60, 80 and 100 mV s^{-1} in 1.0 M KOH for PtNiFeCoCu$_{26}$/C, Pt$_{21}$Ni$_{27}$Fe$_{19}$Co$_{17}$Cu$_{16}$/C and Pt$_{15}$Ni$_{24}$Fe$_{13}$Co$_{13}$Cu$_{35}$/C. (d)-(f) Current density as a function of scan rate derived from (a)-(c) at 1.0 V (vs. RHE) of PtNiFeCoCu$_{26}$/C, Pt$_{21}$Ni$_{27}$Fe$_{19}$Co$_{17}$Cu$_{16}$/C and Pt$_{15}$Ni$_{24}$Fe$_{13}$Co$_{13}$Cu$_{35}$/C.

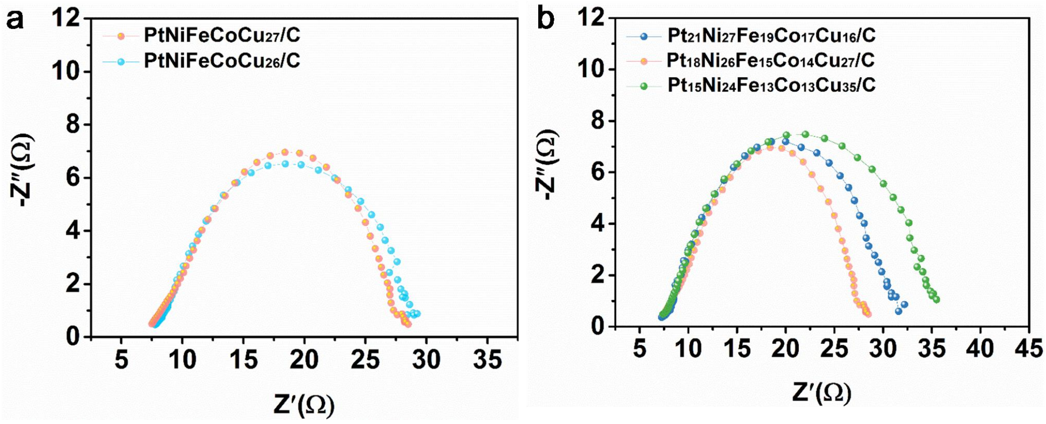

Supplementary Figure 20. The Nyquist plots at -50 mV *vs.* RHE. (a) PtNiFeCoCu$_{26}$/C. (b) Pt$_{21}$Ni$_{27}$Fe$_{19}$Co$_{17}$Cu$_{16}$/C, Pt$_{18}$Ni$_{26}$Fe$_{15}$Co$_{14}$Cu$_{27}$/C and Pt$_{15}$Ni$_{24}$Fe$_{13}$Co$_{13}$Cu$_{35}$/C.

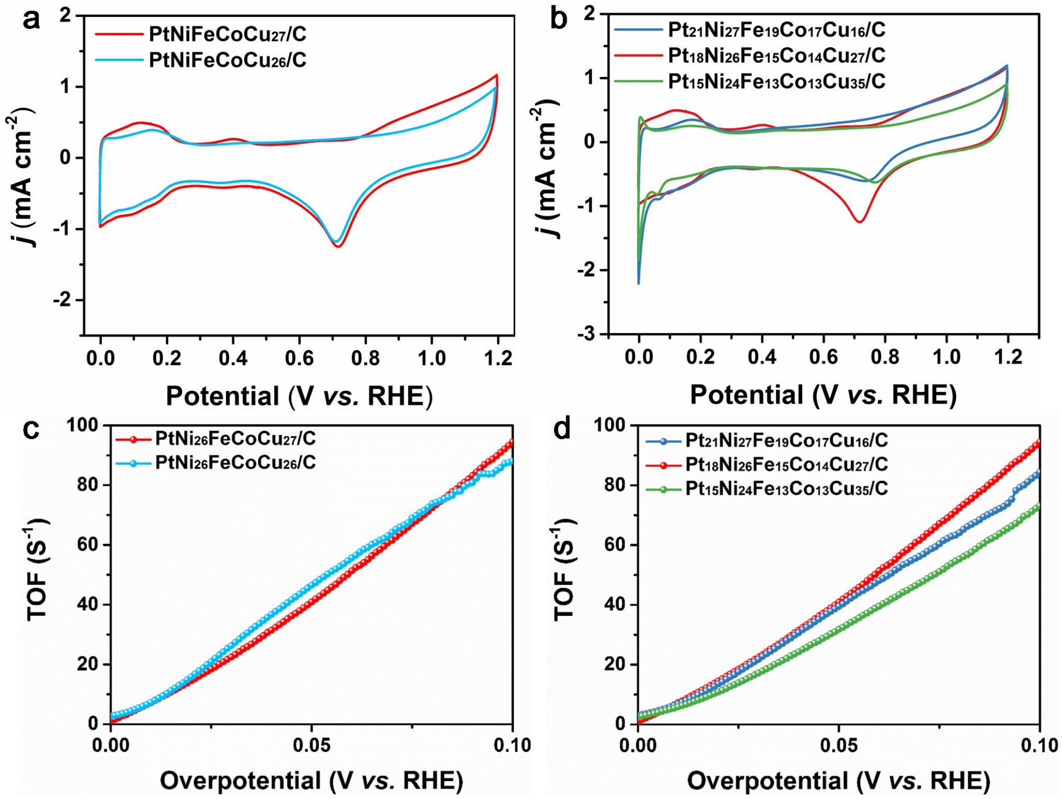

Supplementary Figure 21. Electrocatalytic property evaluation. (a) and (b) CV curves of PtNiFeCoCu/C catalysts in 0.5 M H_2SO_4. (c) and (d) The potential dependent TOF curves of PtNiFeCoCu/C catalysts.

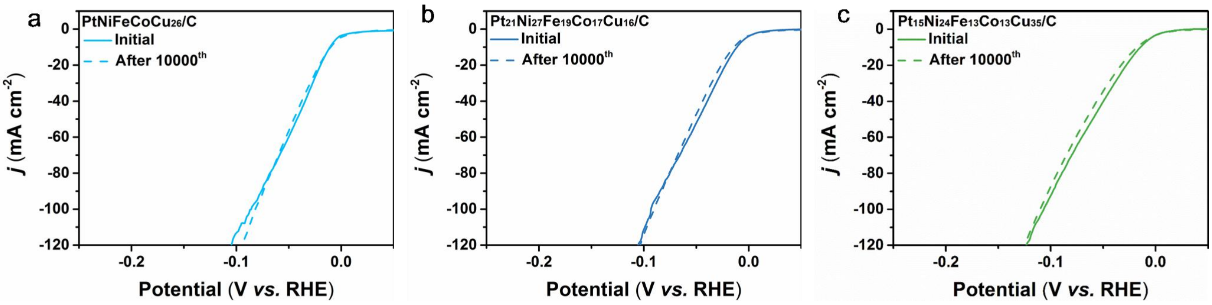

Supplementary Figure 22. Electrocatalytic performance tests. HER polarization curves (geometrical area) for (a) $PtNiFeCoCu_{26}/C$, (b) $Pt_{21}Ni_{27}Fe_{19}Co_{17}Cu_{16}/C$ and (c) $Pt_{15}Ni_{24}Fe_{13}Co_{13}Cu_{35}/C$ before and after 10000 CV cycle.

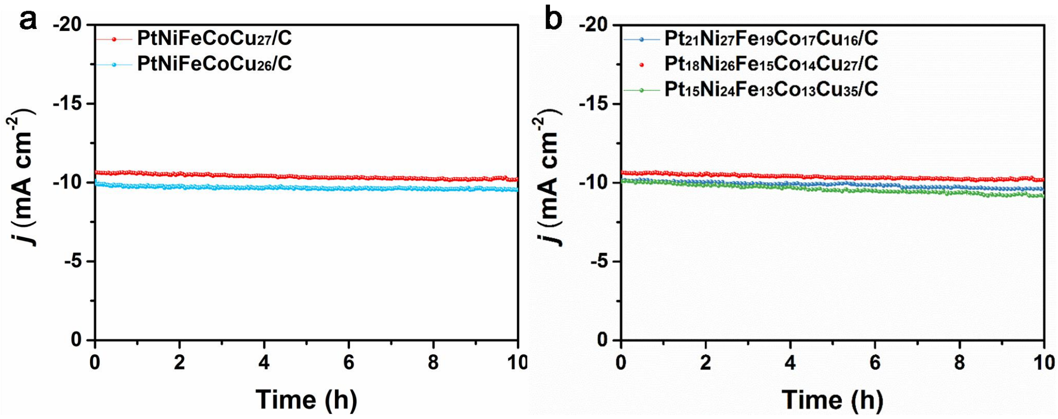

Supplementary Figure 23. Electrocatalytic durability tests for HER. (a) Chronoamperometric measurement curves of $PtNiFeCoCu_{27}$ NPs and $PtNiFeCoCu_{26}$ NPs. (b) Chronoamperometric measurement curves of different PtNiFeCoCu NPs.

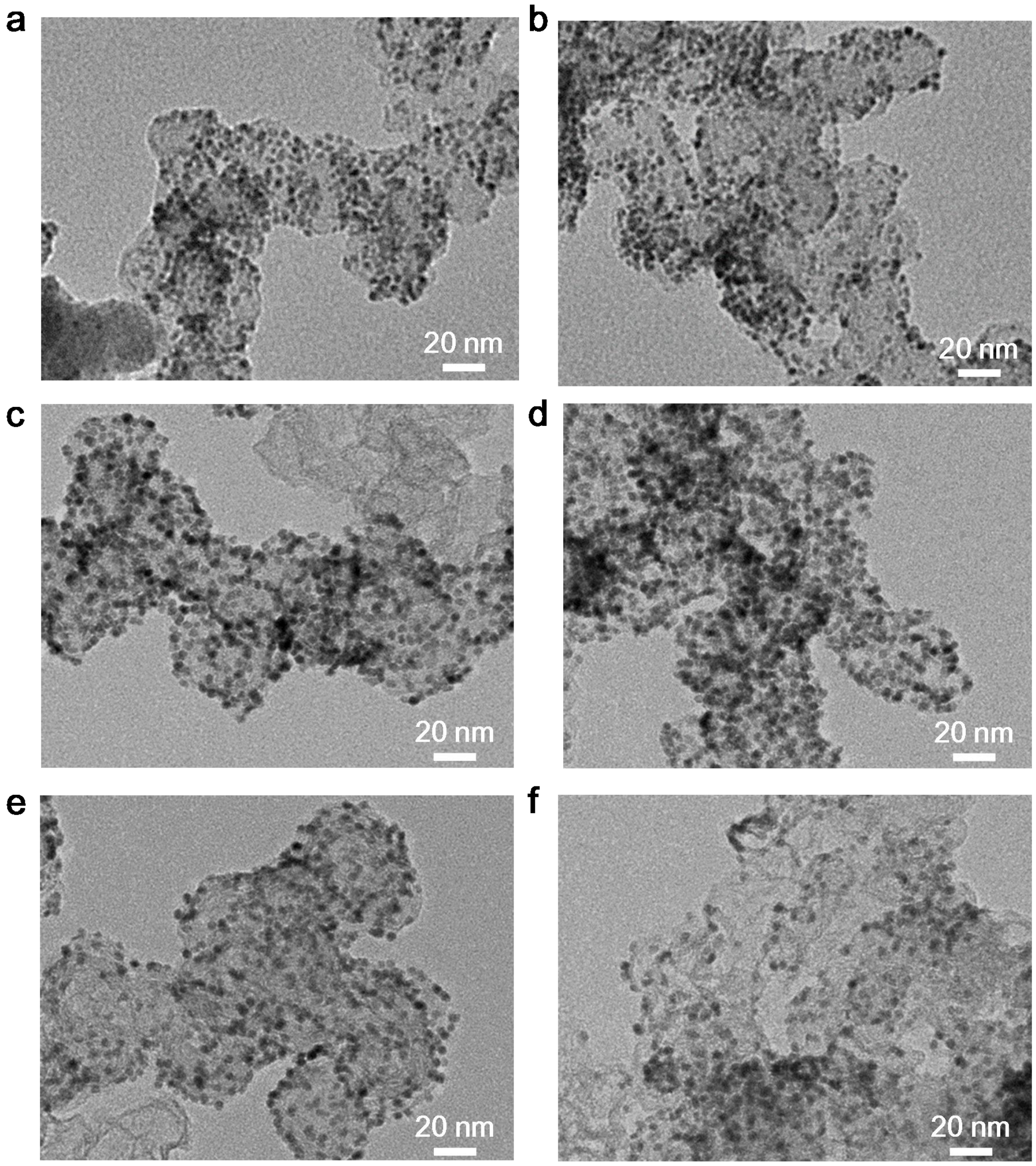

Supplementary Figure 24. TEM images. (a) and (b) The TEM images of $PtNiFeCoCu_{26}/C$. (c) and (d) The TEM images of $Pt_{21}Ni_{27}Fe_{19}Co_{17}Cu_{16}/C$. (e) and (f) The TEM images of $Pt_{15}Ni_{24}Fe_{13}Co_{13}Cu_{35}/C$ before and after stability test for HER.

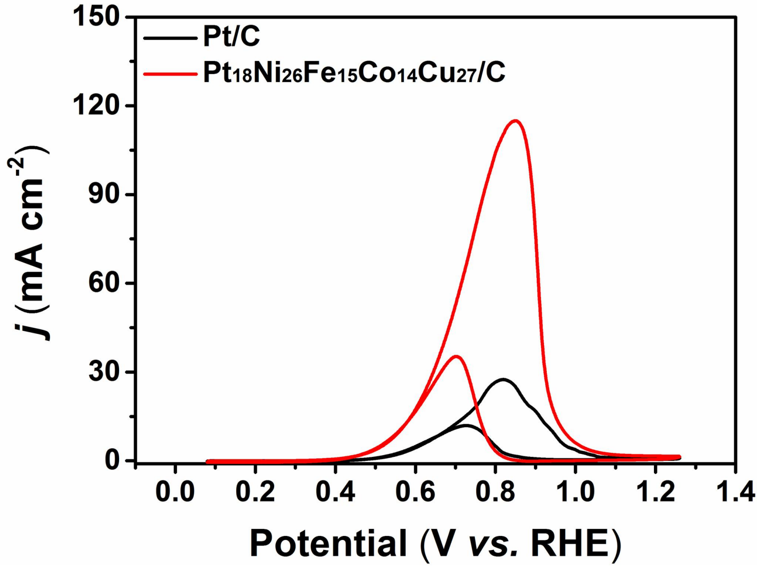

Supplementary Figure 25. The CV curves (geometrical area) of the $Pt_{18}Ni_{26}Fe_{15}Co_{14}Cu_{27}$/C and Pt/C in 1 M KOH + 1 M CH_3OH electrolyte.

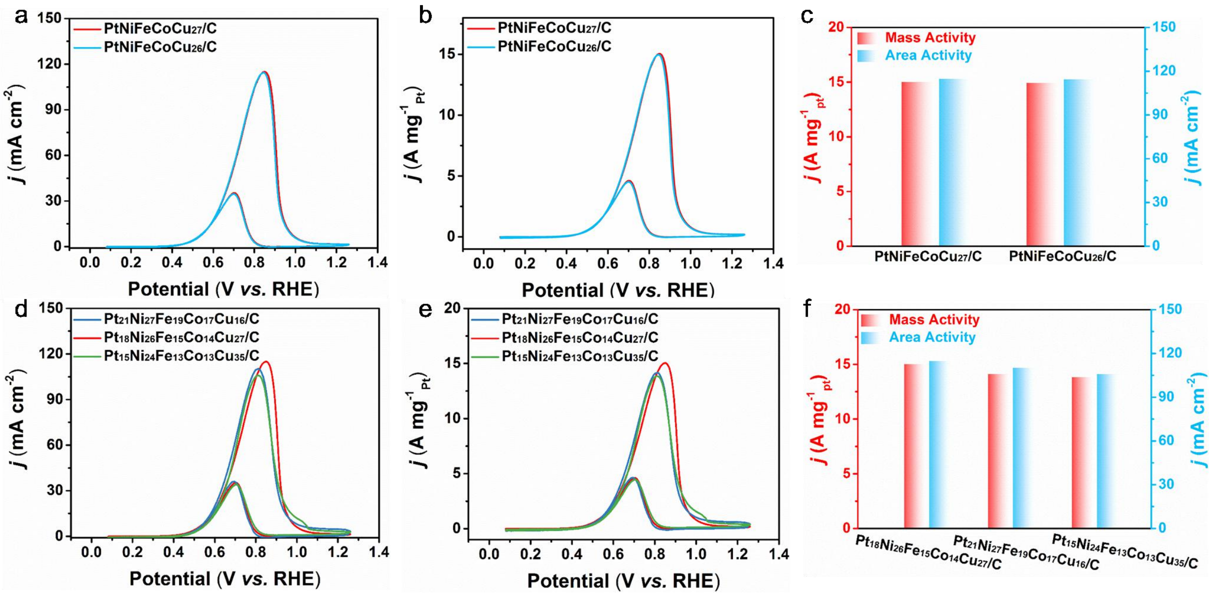

Supplementary Figure 26. Methanol electro-oxidation performance of the PtNiFeCoCu_{26}/C, $Pt_{21}Ni_{27}Fe_{19}Co_{17}Cu_{16}$/C, $Pt_{18}Ni_{26}Fe_{15}Co_{14}Cu_{27}$/C and $Pt_{15}Ni_{24}Fe_{13}Co_{13}Cu_{35}$/C in 1 M KOH + 1 M CH_3OH electrolyte. (a) and (d) CV curves (area activity). (b) and (e) CV curves (mass activity). (c) and (f) Peak values of mass activity and area activity.

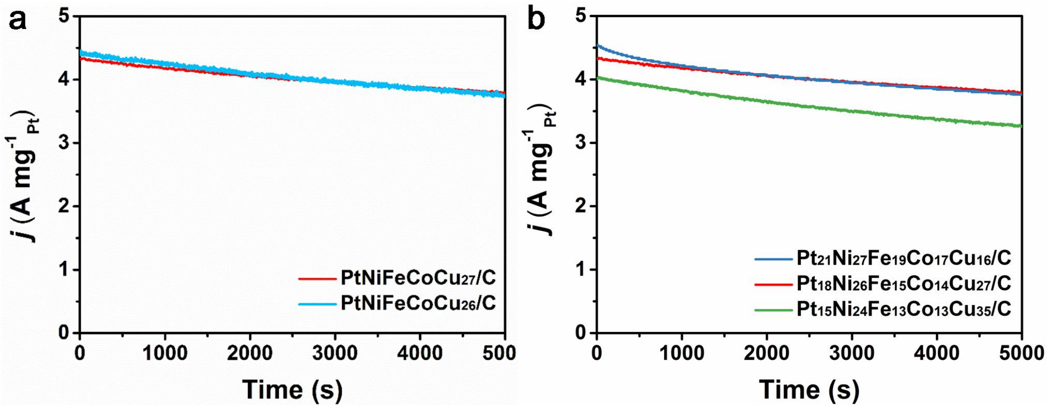

Supplementary Figure 27. Electrocatalytic durability tests for MOR at 0.65 V *vs.* RHE. (a) Chronoamperometric measurement curves of $PtNiFeCoCu_{27}/C$ and $PtNiFeCoCu_{26}/C$ catalysts. (b) Chronoamperometric measurement curves of different PtNiFeCoCu/C catalysts.

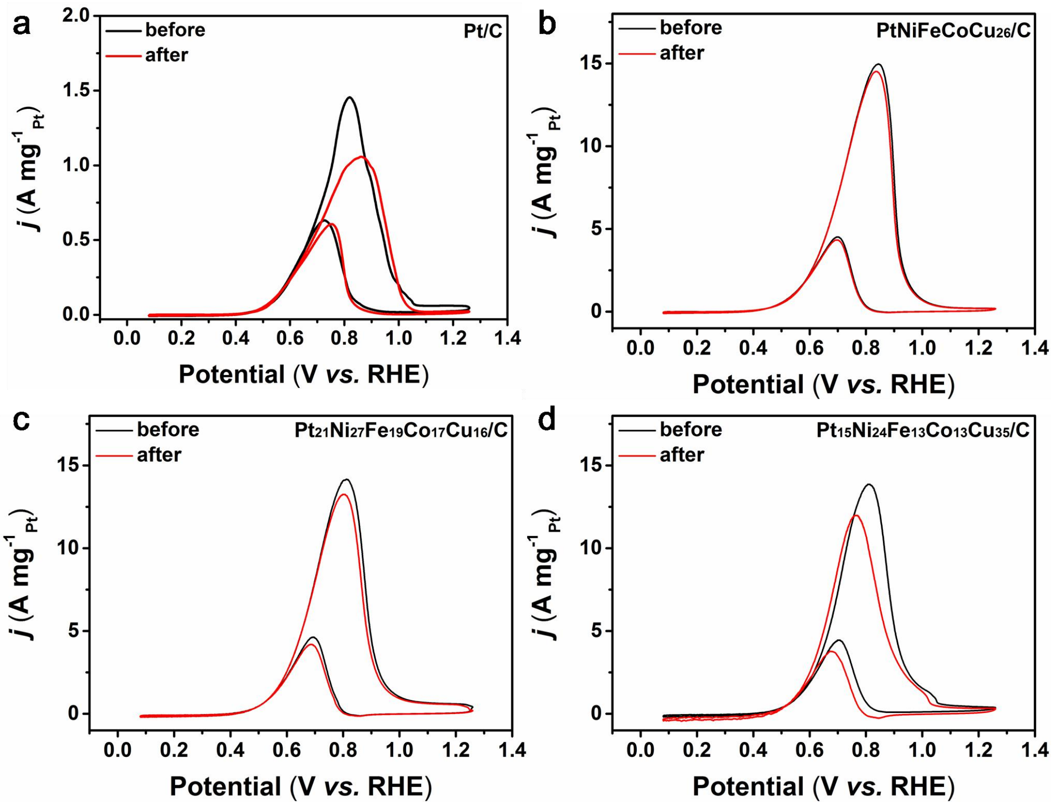

Supplementary Figure 28. Electrocatalytic performance tests. CV curves of the (a) Pt/C, (b) $PtNiFeCoCu_{26}/C$, (c) $Pt_{21}Ni_{27}Fe_{19}Co_{17}Cu_{16}/C$ and (d) $Pt_{15}Ni_{24}Fe_{13}Co_{13}Cu_{35}/C$ before and after 1000 cycles.

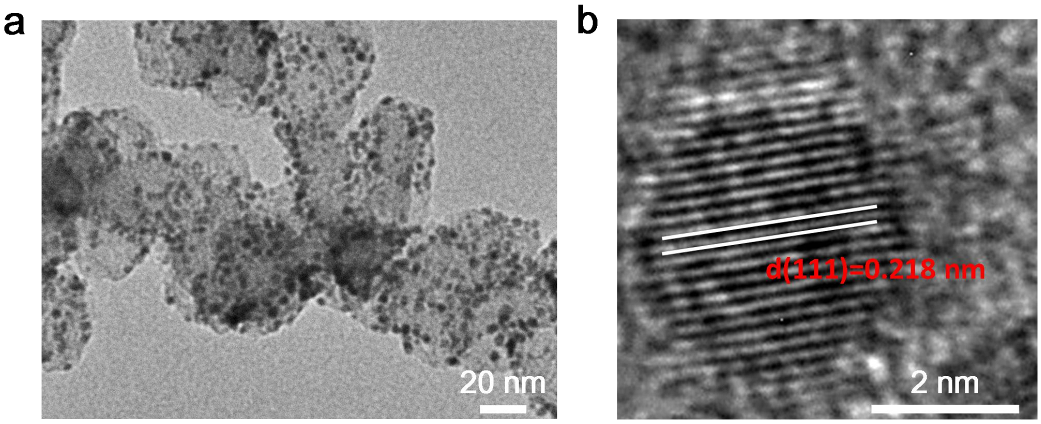

Supplementary Figure 29. TEM images. The (a) TEM image and (b) HRTEM image of $Pt_{18}Ni_{26}Fe_{15}Co_{14}Cu_{27}/C$ after stability test for MOR.

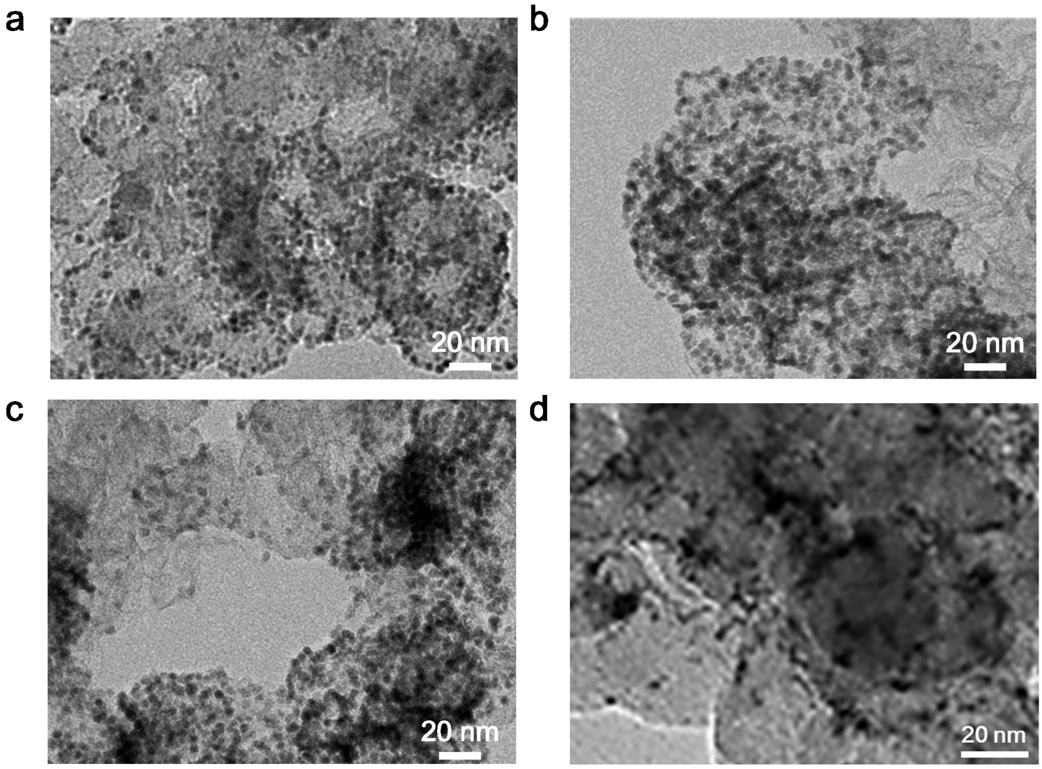

Supplementary Figure 30. TEM images after stability test for MOR. The TEM images of (a) $PtNiFe_{15}CoCu_{26}/C$, (b) $Pt_{21}Ni_{27}Fe_{19}Co_{17}Cu_{16}/C$, (c) $Pt_{15}Ni_{24}Fe_{13}Co_{13}Cu_{35}/C$ and (d) Pt/C.

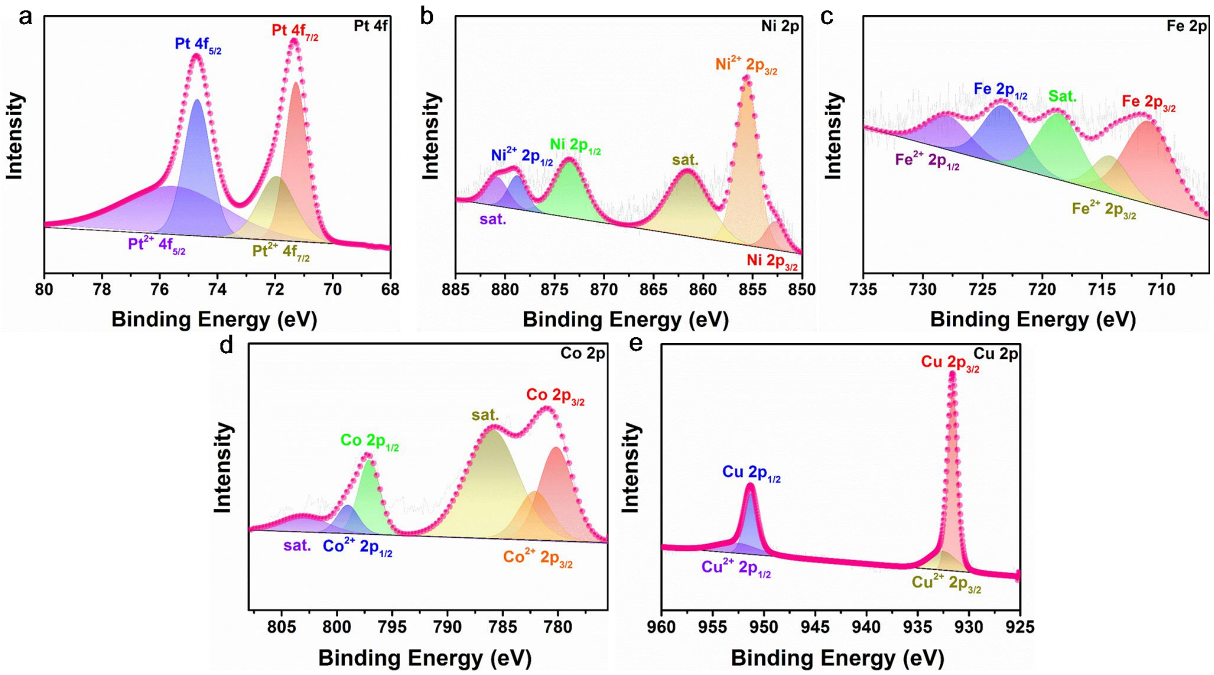

Supplementary Figure 31. XPS analysis of $Pt_{18}Ni_{26}Fe_{15}Co_{14}Cu_{27}/C$ catalyst after MOR. (a) Pt 4f XPS spectrum. (b) Ni 2p XPS spectrum. (c) Fe 2p XPS spectrum. (d) Co 2p XPS spectrum. (e) Cu 2p XPS spectrum.

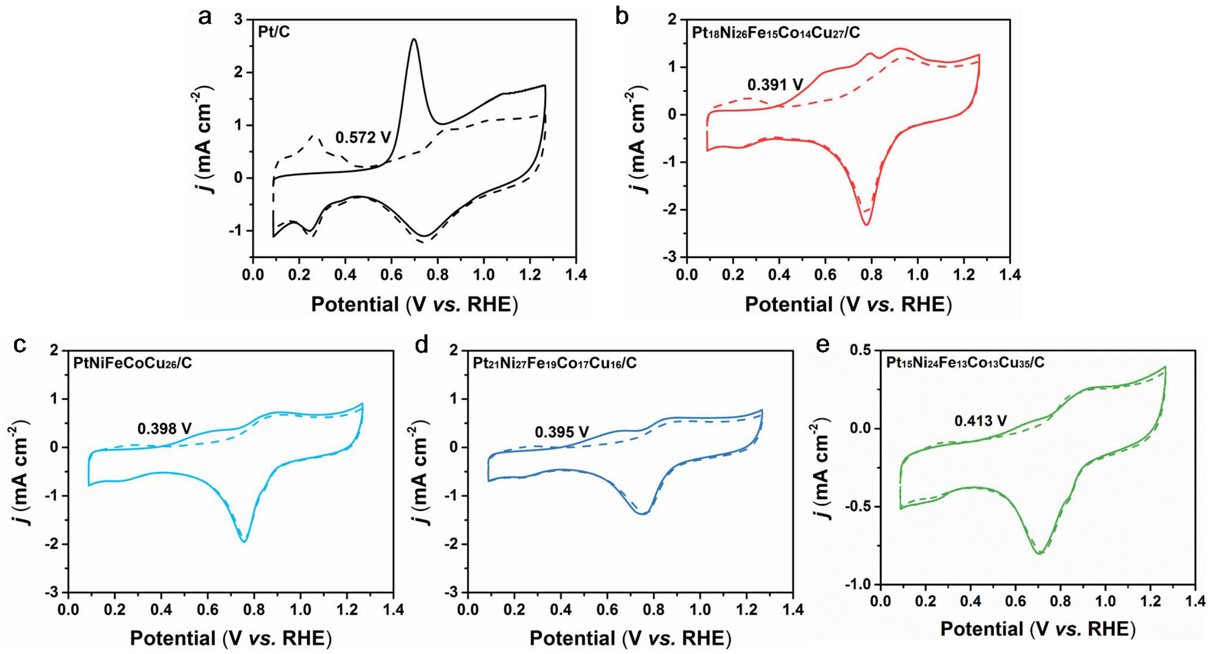

Supplementary Figure 32. CO stripping tests. The CO stripping curves for (a) Pt/C, (b) $Pt_{18}Ni_{26}Fe_{15}Co_{14}Cu_{27}$ NPs/C, (c) $PtNiFe_{15}CoCu_{26}$/C, (d) $Pt_{21}Ni_{27}Fe_{19}Co_{17}Cu_{16}$/C and (e) $Pt_{15}Ni_{24}Fe_{13}Co_{13}Cu_{35}$/C in 1 M KOH electrolyte.

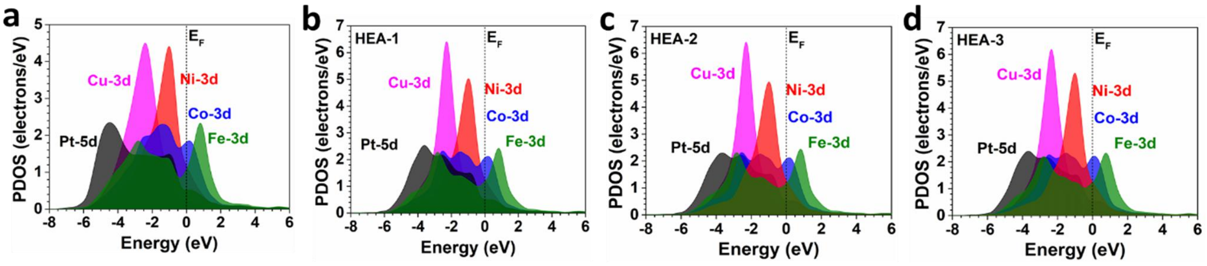

Supplementary Figure 33. The PDOS comparison of the slight stoichiometry variations in HEA structures. (a) The PDOS of applied HEA model in our work. (b)-(d) The PDOS of the different HEA structures with slightly varied stoichiometry.

Supplementary Table 1. Atomic ratios of PtNiFeCoCu NPs characterized by ICP before and after the HER or MOR.

Sample	Pt	Ni	Fe	Co	Cu
initial	18.2	25.8	15.2	13.9	26.9
After HER	19.1	25.7	14.8	13.6	26.8
After MOR	19.4	25.6	14.7	13.7	26.6

Supplementary Table 2. The ICP data (mg L^{-1}) of the electrolyte after electro-catalysis test.

Solution	Pt	Ni	Fe	Co	Cu
After HER	-	0.0043	0.0101	0.0089	0.0052
After MOR	-	0.0067	0.0152	0.0076	0.0086

Supplementary Table 3. The valence state rations of Pt^0/Pt^{2+}, Ni^0/Ni^{2+}, Fe^0/Fe^{2+}, Co^0/Co^{2+}, and Cu^0/Cu^{2+} obtained from XPS spectra of $Pt_{18}Ni_{26}Fe_{15}Co_{14}Cu_{27}/C$ catalyst.

Catalysts	Pt^0/Pt^{2+}	Ni^0/Ni^{2+}	Fe^0/Fe^{2+}	Co^0/Co^{2+}	Cu^0/Cu^{2+}
Initial	1.54	0.19	5.11	1.15	4.45
After HER	1.30	0.15	2.86	1.14	4.14
After MOR	1.52	0.16	3.01	1.11	4.20

Supplementary Table 4. The atomic ratio of Pt, Ni, Fe, Co, and Cu obtained from XPS after Ar^+ sputtering for 10 s.

Catalysts	Pt	Ni	Fe	Co	Cu
Initial	21.2	27.4	15.2	13.7	22.5
After Ar^+ sputtering	21.4	27.1	15.3	13.9	22.3

Supplementary Table 5. Comparing the catalytic performance of PtNiFeCoCu/C with different ratios under alkaline HER catalysts.

Catalysts	Mass Activity at -0.07 V vs. RHE	Area Activity at -0.07 V vs. RHE	$\eta_{10mAcm-2}$ (mV)	Tafel slope (mV dec^{-1})
$Pt_{18}Ni_{26}Fe_{15}Co_{14}Cu_{27}/C$	10.96 A mg^{-1}_{pt}	83.78 mA cm^{-2}	11	30
$PtNiFeCoCu_{26}/C$	10.81 A mg^{-1}_{pt}	82.66 mA cm^{-2}	11	30
$Pt_{21}Ni_{27}Fe_{19}Co_{17}Cu_{16}/C$	9.65 A mg^{-1}_{pt}	74.21 mA cm^{-2}	12	36
$Pt_{15}Ni_{24}Fe_{13}Co_{13}Cu_{35}/C$	7.88 A mg^{-1}_{pt}	59.76 mA cm^{-2}	15	45

Supplementary Table 6. Comparing the catalytic performance of PtNiFeCoCu/C with different ratios under alkaline MOR catalysts.

Catalysts	Mass Activity	Area Activity
$Pt_{18}Ni_{26}Fe_{15}Co_{14}Cu_{27}/C$	15.04 A mg^{-1}_{pt}	114.93 mA cm^{-2}
$PtNiFeCoCu_{26}/C$	14.96 A mg^{-1}_{pt}	114.36 mA cm^{-2}
$Pt_{21}Ni_{27}Fe_{19}Co_{17}Cu_{16}/C$	14.16 A mg^{-1}_{pt}	110.25 mA cm^{-2}
$Pt_{15}Ni_{24}Fe_{13}Co_{13}Cu_{35}/C$	13.85 A mg^{-1}_{pt}	105.87 mA cm^{-2}

Supplementary Table 7. Comparing the catalytic performance of $Pt_{18}Ni_{26}Fe_{15}Co_{14}Cu_{27}/C$ with the ever-reported alkaline HER catalysts.

Catalysts	Electrolyte	Mass Activity	$\eta_{10mA\ cm^{-2}}$ (mV)	References
**$Pt_{18}Ni_{26}Fe_{15}Co_{14}Cu_{27}/C$**	**1.0 M KOH**	**7.89 A mg^{-1}_{pt} at -0.05 V vs. RHE 10.96 A mg^{-1}_{pt} at -0.07 V vs. RHE**	**11**	**This work**
Pt NWs/SL-$Ni(OH)_2$	1.0 M KOH	0.68 A mg^{-1}_{pt} at -0.07 V vs. RHE	~70	1
Pt_3Ni_2 NWs-S/C	1.0 M KOH	2.45 A mg^{-1}_{pt} at -0.07 V vs. RHE	42	2
Hcp-Pt-Ni	0.1 M KOH	3.03 A mg^{-1}_{pt} at -0.07 V vs. RHE	65	3
20 wt% Pt/$Ni(HCO_3)_2$	1.0 M KOH	0.81 A mg^{-1}_{pt} at -0.07 V vs. RHE	44	4
4wt% Pt_c/$Ni(OH)_2$	0.1 M KOH	6.34 A mg^{-1}_{pt} at -0.05 V vs. RHE	32	5
SANi-Pt NWs	1.0 M KOH	11.80 A mg^{-1}_{pt} at -0.07 V vs. RHE	-	6
PtNi-O/C	1.0 M KOH	7.23 A mg^{-1}_{pt} at -0.07 V vs. RHE	40	7
NiO_x/Pt_3Ni NWs	1.0 M KOH	2.59 A mg^{-1}_{pt} at -0.07 V vs. RHE	40	8
Pt-Ni(N)	1.0 M KOH	-	13	9
FeCoNiAlTi-D15h	1.0 M KOH	-	88	10
NiFeMoCoCr HEA	1.0 M KOH	-	172	11
CoFeLaNiPt HEMG-NP	0.1 M KOH	-	555	12

Supplementary Table 8. Comparing the catalytic performance of $Pt_{18}Ni_{26}Fe_{15}Co_{14}Cu_{27}/C$ with the ever-reported alkaline MOR catalysts.

Catalysts	Electrolyte	Mass Activity	References
**$Pt_{18}Ni_{26}Fe_{15}Co_{14}Cu_{27}/C$**	**1 M KOH 1 M MeOH**	**15.04 A mg^{-1}_{Pt}**	**This work**
PtZn intermetallic NPs	0.1 M KOH 0.5 M MeOH	~0.58 A mg^{-1}	13
Pt-$Ni(OH)_2$-rGO	1 M KOH 1 M MeOH	1.23 A mg^{-1}_{Pt}	14
Au/Ag/Pt hetero-nanostructure	1 M KOH 1 M MeOH	~1.00 A mg^{-1}_{Pt}	15
PtAuRu	1 M KOH 1 M MeOH	1.60 A mg^{-1}_{metal}	16
$Pd_3Pb/Pt_{2.37}Pb$ Nanocubes	1 M KOH 1 M MeOH	8.40 A mg^{-1}_{Pt}	17
SANi-Pt NWs	1 M KOH 1 M MeOH	7.93 A mg^{-1}_{Pt}	6
$Pt_{0.5}Ag_1$	0.5 M KOH 2 M MeOH	2.92 A mg^{-1}_{Pt}	18
CS-$Pt_{56}Cu_{28}Ni_{16}$	1 M KOH 1 M MeOH	7.00 A mg^{-1}_{Pt}	19
PtNi/C	1 M NaOH 1 M MeOH	1.20 A mg^{-1}_{Pt}	20
Popcorn-like PtAu	1 M KOH 1 M MeOH	0.60 A mg^{-1}_{Pt}	21
Pt_1Ni_1/C	1 M KOH 1 M MeOH	1.75 A mg^{-1}_{Pt}	22
$Pt_{3.5}Pb$ nerve nanowires	0.5 M KOH 1M MeOH	2.84 A mg^{-1}_{Pt}	23
PtAg popcorns	1 M KOH 1 M MeOH	1.65 A mg^{-1}_{Pt}	24
PtCu nanoframes	0.5 M KOH 1M MeOH	2.26 A mg^{-1}_{Pt}	25
Porous Pt Nanotubes	1 M KOH 1 M MeOH	2.33 A mg^{-1}_{Pt}	26
Pt/rGO	0.5 M KOH 0.5 M MeOH	0.55 A mg^{-1}_{Pt}	27

Supplementary References

1 Yin, H. et al. Ultrathin platinum nanowires grown on single-layered nickel hydroxide with high hydrogen evolution activity. *Nat. Commun.* **6,** 6430 (2015).

2 Wang, P. et al. Precise tuning in platinum-nickel/nickel sulfide interface nanowires for synergistic hydrogen evolution catalysis. *Nat. Commun.* **8,** 14580 (2017).

3 Cao, Z. et al. Platinum-nickel alloy excavated nano-multipods with hexagonal close-packed structure and superior activity towards hydrogen evolution reaction. *Nat. Commun.* **8,** 15131 (2017).

4 Lao, M. et al. Platinum/nickel bicarbonate heterostructures towards accelerated hydrogen evolution under alkaline conditions. *Angew. Chem. Int. Ed.* **58,** 5432-5437 (2019).

5 Yang, H. et al. Atomic-scale Pt clusters decorated on porous α-$Ni(OH)_2$ nanowires as highly efficient electrocatalyst for hydrogen evolution reaction. *Sci. China Mater.* **60,** 1121-1128 (2017).

6 Li, M. et al. Single-atom tailoring of platinum nanocatalysts for high-performance multifunctional electrocatalysis. *Nat. Catal.* **2,** 495-503 (2019).

7 Zhao, Z. et al. Surface-engineered PtNi-O nanostructure with record-high performance for electrocatalytic hydrogen evolution reaction. *J. Am. Chem. Soc.* **140,** 9046-9050 (2018).

8 Wang, P., Jiang, K., Wang, G., Yao, J. & Huang, X. Phase and interface engineering of platinum-nickel nanowires for efficient electrochemical hydrogen evolution. *Angew. Chem. Int. Ed.* **55,** 12859-12863 (2016).

9 Xie, Y. et al. Boosting water dissociation kinetics on Pt-Ni nanowires by N-induced orbital tuning. *Adv. Mater.* **31,** 1807780 (2019).

10 Jia, Z. et al. A novel multinary intermetallic as an active electrocatalyst for hydrogen evolution. *Adv. Mater.* **32,** 2000385 (2020).

11 Zhang, G. et al. High entropy alloy as a highly active and stable electrocatalyst for hydrogen evolution reaction. *Electrochim. Acta* **279,** 19-23 (2018).

12 Glasscott, M. W. et al. Electrosynthesis of high-entropy metallic glass nanoparticles for designer, multi-functional electrocatalysis. *Nat. Commun.* **10,** 2650 (2019).

13 Qi, Z. et al. Sub-4 nm PtZn intermetallic nanoparticles for enhanced mass and specific activities in catalytic electrooxidation reaction. *J. Am. Chem. Soc.* **139,** 4762-4768 (2017).

14 Huang, W. et al. Highly active and durable methanol oxidation electrocatalyst based on the synergy of platinum-nickel hydroxide-graphene. *Nat. Commun.* **6,** 10035 (2015).

15 Xie, X. et al. Site-selective trimetallic heterogeneous nanostructures for enhanced electrocatalytic performance. *Adv. Mater.* **27,** 5573-5577 (2015).

16 Ren, F. et al. One-pot synthesis of a RGO-supported ultrafine ternary PtAuRu catalyst with high electrocatalytic activity towards methanol oxidation in alkaline medium. *J. Mater. Chem. A* **1,** 7255-7261 (2013).

17 Wu, X. et al. Tuning surface structure of Pd_3Pb/Pt_nPb nanocrystals for boosting the methanol oxidation reaction. *Adv. Sci.* **6,** 1902249 (2019).

18 Feng, Y. Y., Bi, L. X., Liu, Z. H., Kong, D. S. & Yu, Z. Y. Significantly enhanced electrocatalytic activity for methanol electro-oxidation on Ag oxide-promoted PtAg/C catalysts in alkaline electrolyte. *J. Catal.* **290,** 18-25 (2012).

19 Huang, J. et al. PtCuNi tetrahedra catalysts with tailored surfaces for efficient alcohol oxidation. *Nano Lett.* **19,** 5431-5436 (2019).

20 Jiang, Q., Jiang, L., Wang, S., Qi, J. & Sun, G. A highly active PtNi/C electrocatalyst for methanol electro-oxidation in alkaline media. *Catal. Commun.* **12,** 67-70 (2010).

21 Zheng, J. N. et al. Popcorn-like PtAu nanoparticles supported on reduced graphene oxide: Facile synthesis and catalytic applications. *J. Mater. Chem. A* **2,** 8386-8395 (2014).

22 Lu, S., Li, H., Sun, J. & Zhuang, Z. Promoting the methanol oxidation catalytic activity by introducing surface nickel on platinum nanoparticles. *Nano Res.* **11,** 2058-2068 (2018).

23 Huang, L., Han, Y., Zhang, X., Fang, Y. & Dong, S. One-step synthesis of ultrathin Pt_xPb nerve-like nanowires as robust catalysts for enhanced methanol electrooxidation. *Nanoscale* **9,** 201-207 (2017).

24 Zhang, W., Yang, J. & Lu, X. Tailoring galvanic replacement reaction for the preparation of Pt/Ag bimetallic hollow nanostructures with controlled number of voids. *ACS Nano* **6,** 7397-7405 (2012).

25 Zhang, Z. et al. One-pot synthesis of highly anisotropic five-fold-twinned PtCu nanoframes used as a bifunctional electrocatalyst for oxygen reduction and methanol oxidation. *Adv. Mater.* **28,** 8712-8717 (2016).

26 Lou, Y. et al. Porous Pt nanotubes with high methanol oxidation electrocatalytic activity based on original bamboo-shaped Te nanotubes. *ACS Appl. Mater. Inter.* **8,** 16147-16153 (2016).

27 Wu, S. et al. Highly dispersed ultrafine Pt nanoparticles on reduced graphene oxide nanosheets: In situ sacrificial template synthesis and superior electrocatalytic performance for methanol oxidation. *ACS Appl. Mater. Inter.* **7,** 22935-22940 (2015).
